# Expansion of Activated Peripheral Blood Memory B Cells in Rheumatoid Arthritis, Impact of B Cell Depletion Therapy, and Biomarkers of Response

**DOI:** 10.1371/journal.pone.0128269

**Published:** 2015-06-05

**Authors:** Diana G. Adlowitz, Jennifer Barnard, Jamie N. Biear, Christopher Cistrone, Teresa Owen, Wensheng Wang, Arumugam Palanichamy, Ezinma Ezealah, Debbie Campbell, Chungwen Wei, R. John Looney, Inaki Sanz, Jennifer H. Anolik

**Affiliations:** 1 Department of Medicine, Division of Allergy, Immunology and Rheumatology, University of Rochester Medical Center, Rochester, New York, 14642, United States of America; 2 Department of Medicine, Emory University, Atlanta, Georgia, 30332, United States of America; University Hospital Jena, GERMANY

## Abstract

Although B cell depletion therapy (BCDT) is effective in a subset of rheumatoid arthritis (RA) patients, both mechanisms and biomarkers of response are poorly defined. Here we characterized abnormalities in B cell populations in RA and the impact of BCDT in order to elucidate B cell roles in the disease and response biomarkers. In active RA patients both CD27+IgD- switched memory (SM) and CD27-IgD- double negative memory (DN) peripheral blood B cells contained significantly higher fractions of CD95+ and CD21- activated cells compared to healthy controls. After BCD the predominant B cell populations were memory, and residual memory B cells displayed a high fraction of CD21- and CD95+ compared to pre-depletion indicating some resistance of these activated populations to anti-CD20. The residual memory populations also expressed more Ki-67 compared to pre-treatment, suggesting homeostatic proliferation in the B cell depleted state. Biomarkers of clinical response included lower CD95+ activated memory B cells at depletion time points and a higher ratio of transitional B cells to memory at reconstitution. B cell function in terms of cytokine secretion was dependent on B cell subset and changed with BCD. Thus, SM B cells produced pro-inflammatory (TNF) over regulatory (IL10) cytokines as compared to naïve/transitional. Notably, B cell TNF production decreased after BCDT and reconstitution compared to untreated RA. Our results support the hypothesis that the clinical and immunological outcome of BCDT depends on the relative balance of protective and pathogenic B cell subsets established after B cell depletion and repopulation.

## Introduction

Rheumatoid arthritis (RA) is a chronic autoimmune disease [1, 2] associated with aggressive synovitis that over time causes bone, tendon, and cartilage damage. Although multiple cell types play a role in the pathogenesis of RA, the key participation of B cells has long been appreciated since the discovery of rheumatoid factor (RF) and has been re-highlighted over the past several years. Thus, RF and anti-cyclic-citrillunated peptide (anti-CCP) autoantibodies are well-established indicators of disease and disease severity and may precede the onset of disease by many years [3–5]. Although B cells have been considered important as producers of autoantibodies, their antibody independent roles and utility as a major therapeutic target have not been appreciated until more recently.

The efficacy of B cell depletion therapy (BCDT) highlights the pathogenic significance of B cells in RA [6–8]. Moreover, the dissociation between changes in autoantibodies and clinical efficacy points to the autoantibody independent roles of B cells in the disease. These may include antigen-presentation, T-cell activation/polarization, dendritic cell modulation, and formation of ectopic lymphoid structures [9–11] [12] and are mediated at least in part by the ability of B cells to produce cytokines [13]. However, the precise contribution of B cells to the disease process and in turn the mechanism(s) by which BCDT is efficacious in RA remain incompletely elucidated. B cells can contribute to autoimmunity via the secretion of pro-inflammatory cytokines such as TNF-α and IL-6 [14, 15], but also may play a protective or regulatory role in the immune system likely depending on the particular subset and inflammatory milieu [16–18]. Recent provocative data in a multiple sclerosis murine model suggests that IL6 producing B cells contribute to T cell stimulation in the disease, including Th17 polarization, and BCDT ameliorated the disease only in mice with IL6-sufficient B cells. Notably, B cells from multiple sclerosis (MS) patients also produced more IL6, an abnormality that was normalized with B cell reconstitution after rituximab [19]. Given that the B cells reemerging after BCDT are dominated by CD27- naïve/transitional cells [20, 21], it is tempting to speculate that the cytokine normalization is related to a shift in the predominant B cell subsets present. However, which B cell subsets produce pro-inflammatory cytokines in RA, the contribution of B cell protective functions, and the potential plasticity of B cell function depending on environmental context remains unknown.

We have previously described that a B cell reconstitution with naïve/transitional cells is associated with sustained clinical remission in systemic lupus erythematosus (SLE) while a quick resurgence of memory cells portends a poor outcome [22, 23]. A number of publications have also found in RA that the detection of residual peripheral blood B cells using high sensitivity flow and the return of B cells, especially with higher fractions of memory B cells, increases the risk of inadequate response and/or relapse [24] [21, 25]. However, a critical question that remains to be addressed is whether the benefit of BCDT is directly mediated by the expanded transitional cells (a putative regulatory B cell subset [18, 26]) or instead reflects the absence of pro-inflammatory B cells or a combination of both. In this study, we followed RA patients longitudinally as they began BCDT in an effort to define the factors that regulate BCD and reconstitution and whether there are biomarkers that may predict response prior to or early after treatment. Of note, we find that active RA at baseline is associated with an activated peripheral blood B cell memory compartment. These memory B cell populations became dominant at depletion time points and displayed evidence of recent cell cycle entry, suggesting a resistance to depletion and homeostatic proliferation. Residual activated memory B cells at depletion time points correlated with incomplete clinical response. B cell repopulation began by 8 months in most patients regardless of response, but a higher ratio of transitional to memory B cells correlated with better clinical outcomes. Memory B cells had a greater propensity to produce pro-inflammatory cytokines particularly TNF, and B cell TNF production was reduced after depletion and reconstitution. These results suggest that the outcome of B cell depletion therapy depends on the relative balance of protective and pathogenic B cell subsets established after B cell depletion and upon B cell repopulation.

## Methods

### Patient Population

Patients met ACR 1987 revised criteria for the classification of RA and had active disease such that rituximab (1000 mg x 2 two weeks apart) was being initiated clinically (see Table 1 for clinical characteristics of patients) [27]. Three of the patients had previously been treated with rituximab and were being re-treated because of disease flare. The mean baseline DAS28 (CRP) score was 4.94 (range 2.99 to 7.19) for the entire group, 4.97 for the rituximab naïve and 4.72 for the re-treated patients. Patients were clinically evaluated and blood was drawn for processing at baseline, 1, 2, 4, 6, 8, 12, 16, and 20 months. The DAS response was determined at 4 months as follows: a decrease in the DAS response of ≥ 1.2 and a DAS ≤ 3.2 was considered a good responder, a decrease in DAS of < 0.6 or 0.6 to 1.2 with a DAS > 5.1 was considered a non-responder, and a moderate responder was between the two groups.

**Table 1:** Clinical characteristics of RA patients.

	RA patients
	(n = 20)
Age, mean ± SD years	62.3 ± 11.5
Women, no. (%)	17 (85)
Disease duration, mean ± SD years	18.8 ± 12.9
Baseline DAS*, mean ± SD	4.9 ± 1.2
RF+ (%)	15 (75)
CCP + (%)	16 (80)
Erosive + (%)	15 (75)
Steroid use (%)	6 (30)
MTX use during treatment (%)	12 (60)
Previous anti-TNF (%)	16 (80)
Number of Previous anti-TNF, mean ± SD (range)	1.6 ± 0.9 (0–3)
Rituximab naïve no. (%)	17 (85)
Time since last Rituximab treatment, range (month)	9–22

*DAS28 (CRP)

### Ethics Statement

Subjects were recruited at the University of Rochester Medical Center (URMC). Detailed written informed consent was obtained from all patients (n = 20) and healthy donors (n = 24) in accordance with a protocol specifically approved by the Human Subjects Institutional Review Board (IRB) of URMC for this study (Protocol RSRB #33286).

### PBMC isolation and Flow Cytometry

Peripheral blood mononuclear cells (PBMCs) were isolated from heparinized blood by Ficoll-Hypaque density gradient centrifugation (Pharmacia Biotech, Uppsala, Sweden). Immunofluorescence staining for flow cytometric analysis was performed by incubating PBMCs with optimally titrated concentrations of mAb in PBS/0.5% BSA on ice for 20 minutes after blocking with 10 μg of human IgG for 20 minutes. Cells were washed in PBS/BSA and incubated with streptavidin-conjugate, Aqua Live/Dead Stain (1:1000), and fixed in 0.5% Formaldehyde before analysis on a LSRII Cytometer with a three-laser (Blue/Red/Violet lasers) configuration (Becton-Dickinson, Mountain View, CA). We used 2 different panels of 12-color/14-parameter flow cytometry with well-validated Standard Operating Procedures (SOPs) that incorporate extensive Quality Assessment and Quality Controls as previously described [28]. These multicolor panels share seven anchor markers including antibodies against CD19 and CD3, along with the Fixable Aqua Dead Cell Stain, to allow the unambiguous identification of live CD19+CD3- B cells. The inclusion of four developmental markers (IgD, CD24, CD27 and CD38) in the same panel makes it feasible to compare and integrate different B cell classification schemes and provide precise identification of the core human B cell subpopulations as further described below. Beads were used as compensation controls (Simply Cellular Compensation Standard, Bangs Laboratories) and FMO controls (Fluorescent minus one) were utilized to define positive expression. Application-specific PMT settings for the LSRII Cytometer were determined for each panel to allow for maximal separation of signal from background and minimal spillover from other fluorochromes into each detector. In addition to the daily instrument calibration with CST beads (BD), the Rainbow Calibration Peak 6 Particles (Spherotech) was run through the Cytometer before each data acquisition to ensure proper instrument performance.

Lymphocytes were gated through the FSC-A vs SSC-A plot and further interrogated by the ratios of Height to Width in forward scatter and side scatter. B cells were identified based on CD19 expression, exclusion of CD3, and gating out cell aggregates and dead cells. Naïve B cells were distinguished from transitional cells and memory B cells by the expression of ABCB1 transporter activity and Mitotracker dye extrusion as described [29]. Briefly, cells were stained in culture medium at 37°C with Mitotracker at 20 nM and chased for 30 minutes prior to flow cytometry analysis. Transitional B cells within the gated IgD+CD27- population were distinguished by the intermediate/high expression of Mitotracker. Additional delineation of early T1/T2 and later T3 transitional populations was based on the relative expression of CD38 and CD24 compared to precursor bone marrow B cell populations as previously described [23]. The incorporation of CD21 and CD95 in the memory panel can provide additional information regarding the activation status of both the CD27+ switched memory (SM) and CD27- memory B cells. Thus, loss of CD21 and up-regulation of CD95 have been independently associated with memory B cell activation [30, 31]. B cells in PBMCs were additionally classified by multi-parameter flow cytometry along a developmental pathway based on the expression of defined surface markers as follows: early transitional (IgD- CD27- CD24+++CD38+++), Transitional 1/2 (IgD+ CD27- MTG+ CD24++/+++CD38++/+++), Transitional 3 (IgD+ CD27- MTG+ CD24+ CD38+), naive (IgD+ CD27- MTG- CD24+CD38+), CD27+ switched memory (IgD- CD27+), CD27- switched memory (IgD- CD27-) (DN), and unswitched memory (IgD+ CD27+). Plasma cells/blasts were included in the CD27+ switched memory compartment as further defined in the figure legends [6, 32–35].

For B cell flow cytometry, 5 million cells were stained with all events collected (minimum of 500,000 events) in order to provide a high sensitivity analysis with the lower limit of detection 0.1 cells/μl [24, 36]. Absolute cell numbers were calculated based on the white blood cell count, the percentage of lymphocytes, and the percentage of CD19 cells identified on flow cytometry. B cell depletion and reconstitution were defined as < or > 5 cells/μl, respectively.

### Sort purification

B cell were enriched from peripheral blood using RosetteSep (Stem Cell Technology), stained with CD19, CD27, IgD and mitotracker, and sorted into unswitched memory (IgD+CD27+), switched memory (IgD-CD27+), mature naïve (IgD+CD27-MTG-), and transitional (IgD+CD27-MTG+) CD19+ B cells (13-color FACSAria-liu, BD Biosciences).

### Cytokine Assays and Ki67 expression

Sorted B cells (100,000) were cultured with 2.5 μg/ml CPG 2006, 2.5 μg/ml anti-CD40, and 50 U/ml IL-2. After 4 days, the B cells were stimulated with 50 ng/ml PMA, 1 μg/ml Ionomycin, and 0.5 μg/ml of Golgi Plug for 4.5 hours. Cells were stained including for intracellular expression of TNF and IL10 and analyzed on a three-laser 12-color LSRII (BD Biosciences).

In other experiments, PBMCs were aliquoted into 1 million per 100 **μ**l of RPMI and stimulated with 200 ng/ml PMA and 2 **μ**g/ml Ionomycin with the addition of 1 μl/ml Golgi Plug and 0.68 μl/ml Golgi Stop at 37°C for 4 hours. The PBMCs were surfaced stained for CD19 and followed with live dead staining. Cells were fixed and internally stained for TNF, IL-17, IFN-γ IL2, IL6, IL-10, CD3, CD4, and Foxp3 (eBioscience). Cells were analyzed on a three-laser 12-color LSRII (BD Biosciences).

Unstimulated PBMCs were surface stained for IgD, CD24, CD21, CD38, CD19, CD20, CD27, CD95, and CD3, followed by live/dead stain, fixation, and antibody against Ki-67. Cells were analyzed on a three-laser 12-color LSRII (BD Biosciences).

### Statistical analysis

The non-parametric Mann-Whitney test was used to compare mean values between pairs of groups. Three group comparisons were conducted using non-parametric ANOVA (Kruskal-Wallis). Paired t-test was performed on paired samples after testing for normality. All tests were two-sided and P values ≤ 0.05 were considered statistically significant. Statistical analyses were performed using Prism software.

## Results

### Expansion of memory B cell populations in active RA

**Fig 1:**
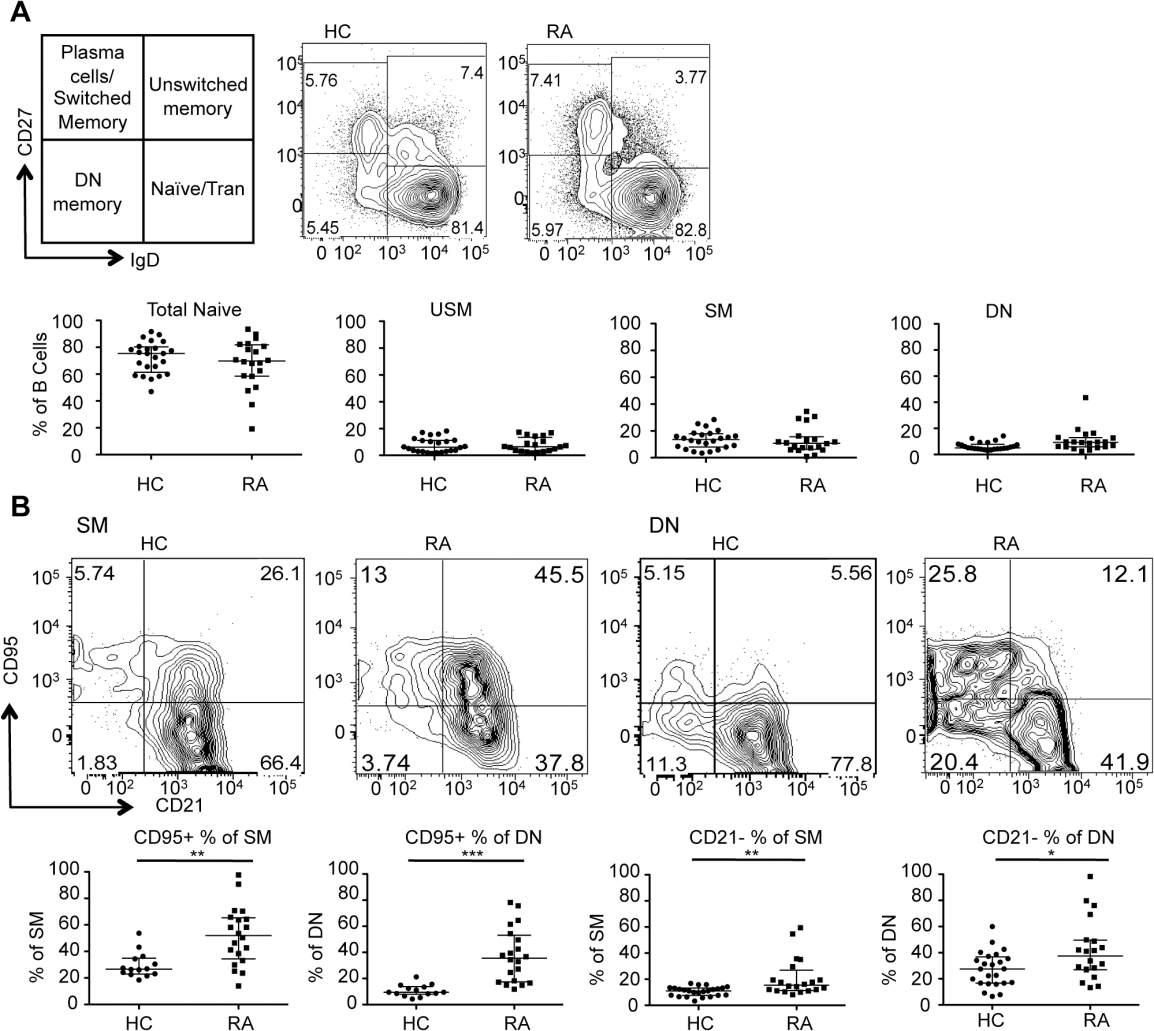
Expansion of activated memory B cells in active RA patients. (A) Multicolor flow cytometry of PBMCs from healthy controls (n = 24) and untreated RA (n = 16) reveals a similar distribution of core total naïve (IgD+CD27-: includes transitional B cells), unswitched memory (USM) (IgD+CD27+), switched memory (SM) (IgD-CD27+: includes plasmablasts) and double negative (DN) memory (IgD-CD27-) B cells. CD19+ B cells gated B cells are shown. (B) The expression of CD95 (up-regulated during activation) and CD21 (down-regulated during activation) on each of the SM and DN memory B cells is defined. The CD95+ B cells from both the SM and DN are significantly higher in active RA before rituximab. There is also a significantly higher representation of CD21 negative B cells in the SM and DN subpopulations in baseline RA (Mann-Whitney) (*p<0.05, **p<0.005, ***p<0.0005). Data is expressed as the median +/- interquartile range.

As an initial analysis of the B cell compartment, we first examined the four canonical B cell subsets revealed by CD27 and IgD expression. When RA pre-BCDT were compared to age-matched healthy controls, the total naïve (IgD+CD27-) (includes transitional B cells), unswitched memory (IgD+CD27+), CD27+ switched memory (IgD-CD27+) (includes plasmablasts), and CD27- DN memory (IgD-CD27-) subsets were similar (Fig 1A). However, notably there were significantly more activated B cell populations in RA as revealed by up-regulation of CD95 and down-regulation of CD21 (Fig 1B). Thus, the CD95+ populations in the SM and DN were significantly higher in the RA patients (p = 0.0020 and p<0.0001, respectively). The CD21- populations in the SM and DN were also significantly higher (p = 0.0010 and p = 0.0392, respectively). Therefore, activated CD95+ and CD21- memory B cells were expanded in active RA compared to age-matched healthy controls.

### Memory B cells predominate initially following rituximab treatment

**Fig 2:**
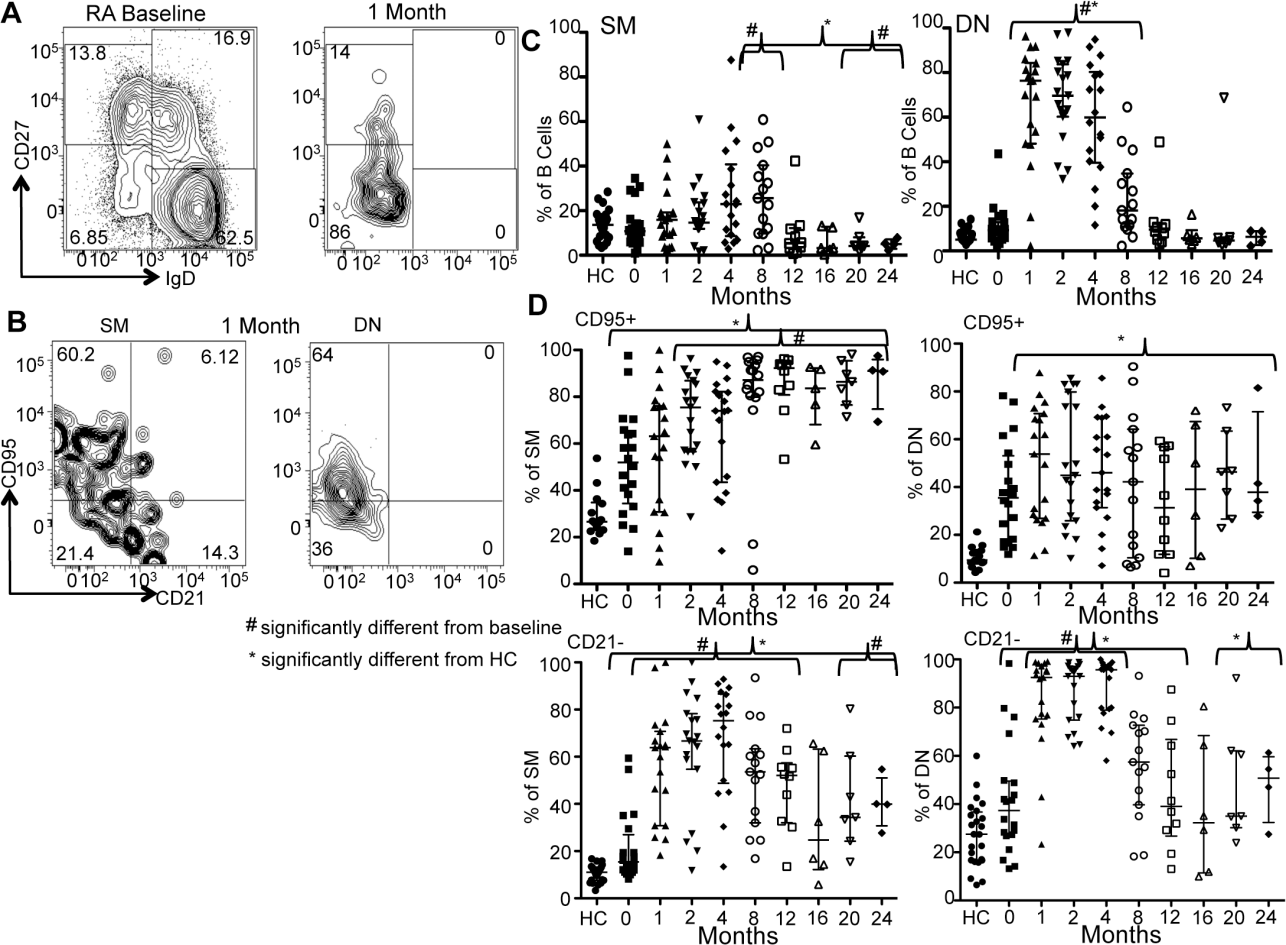
Memory B cell populations become dominant following rituximab treatment. Dot plots show a comparison of B cell subsets in RA at baseline and 1-month post-BCDT for the canonical B cell subsets (A) and (B) the expansion of CD95+ and CD21- B cells in the SM and DN memory B cell populations 1 month after BCDT. (C) The figures depict the change in SM and DN memory over time after BCDT for all subjects. The percentage of both SM and DN B cell populations increase in the earlier months after BCDT as compared to both healthy control and baseline. Percentage of SM B cells subsequently decreases beginning at 12 month. The percentage of DN began to decrease at 8 months. (D) Kinetics of change in the cohort as a whole for CD95 and CD21 expression. *significantly different from HC; # significantly different from baseline p values ranging from <0.05 to <0.0005 (Mann-Whitney). Data is expressed as the median +/- interquartile range.

After BCDT, all subjects depleted to <5 cells/μl (lower limit of detection 0.1 cells/ μl). At depletion time points, the circulating residual B cell populations were predominately of a memory phenotype (IgD-: DN and SM). An example of this finding is depicted in Fig 2A for a typical subject. For the group as a whole, as early as 1 month following BCDT the DN B cell population was relatively expanded compared to both control and baseline and remained so until after B cell reconstitution (healthy control vs. 1–8 months p<0.0001; RA baseline vs. 1–4 months p<0.0001 and 8 month p = 0.0049) (Fig 2C). From 1 to 8 months post-BCDT the relative SM B cell population progressively increased (8 month p = 0.0315 compared to RA baseline) (Fig 2C). Interestingly, once B cell reconstitution was underway (defined as >5 cells/μl and discussed in more detail later) the overall SM B cells were significantly lower than baseline (%SM: 20 months p = 0.0434 and 24 months p = 0.0401) (Fig 2C) (absolute SM baseline 12.39±11.61 cells/μl vs. 1 month nadir 0.25±0.52 cells/μl vs. 20 months 4.5 ± 0.7 cells/μl and 24 month 3.69±1.80 cells/μl). Although absolute numbers of SM and DN did decrease after BCD, there were significant residual B cells (especially DN) compared to the naïve compartment (S1 Fig).

The baseline expansion in activated B cell populations became even more pronounced after BCDT in both the DN and SM (Fig 2B). The CD95+ subset of SM B cells remained significantly higher than controls throughout the duration of the study (to month 24) (Fig 2D). Curiously, during B cell reconstitution (2 months on), this population expanded further and remained higher through 24 months (significantly higher than baseline %: 2 month p = 0.0110, 4 month p<0.0001, 8 month p = 0.0015, 12 month p = 0.0007, 16 month p = 0.0132, 20 month p = 0.0012, and 24 months p = 0.0118) (absolute CD95+ SM baseline 5.65±3.86 cells/μl vs. 1 month nadir 1.43±5.56 cells/μl vs. 16 months 1.92±1.55 cells/μl vs. 20 months 2.15±1.42 cells/μl and 24 months 3.12±1.38). The fraction of CD95+ B cells in the DN population remained higher than controls through 24 months post-BCD. Interestingly, the CD21- expansion within the SM and DN B cells became more pronounced immediately after BCD and persisted through B cell reconstitution (SM: significantly higher than controls through 24 months, months 1–12 p<0.0001, 16 month p = 0.0156, 20 month p = 0.0001 and significantly higher than baseline at months 1–8 p<0.0001 and 12 month p = 0.0017 and again at months 20 p = 0.0138 and 24 months 0.0223) (DN: significantly higher than controls through 12 months, months 1–8 p<0.0001, months 12 p = 0.0472 and significantly higher than baseline at months 1–4 p<0.0001) (Fig 2D). Overall, this data suggested that memory B cells were relatively more resistant to BCD compared to other B cell populations. This is also reflected in the analysis of absolute numbers of cells where activated memory B cell depletion is much less robust compared to the naïve B cell compartment (S1 Fig). The differences in the kinetics of depletion and reconstitution of discrete memory B cell subsets (SM vs. DN and CD95+ vs. CD21-) is interesting and may have multiple explanations including differences in pathways of generation, re-circulation kinetics, and homeostatic proliferation.

### Residual memory B cells in RA following BCDT express the proliferation antigen Ki-67

**Fig 3:**
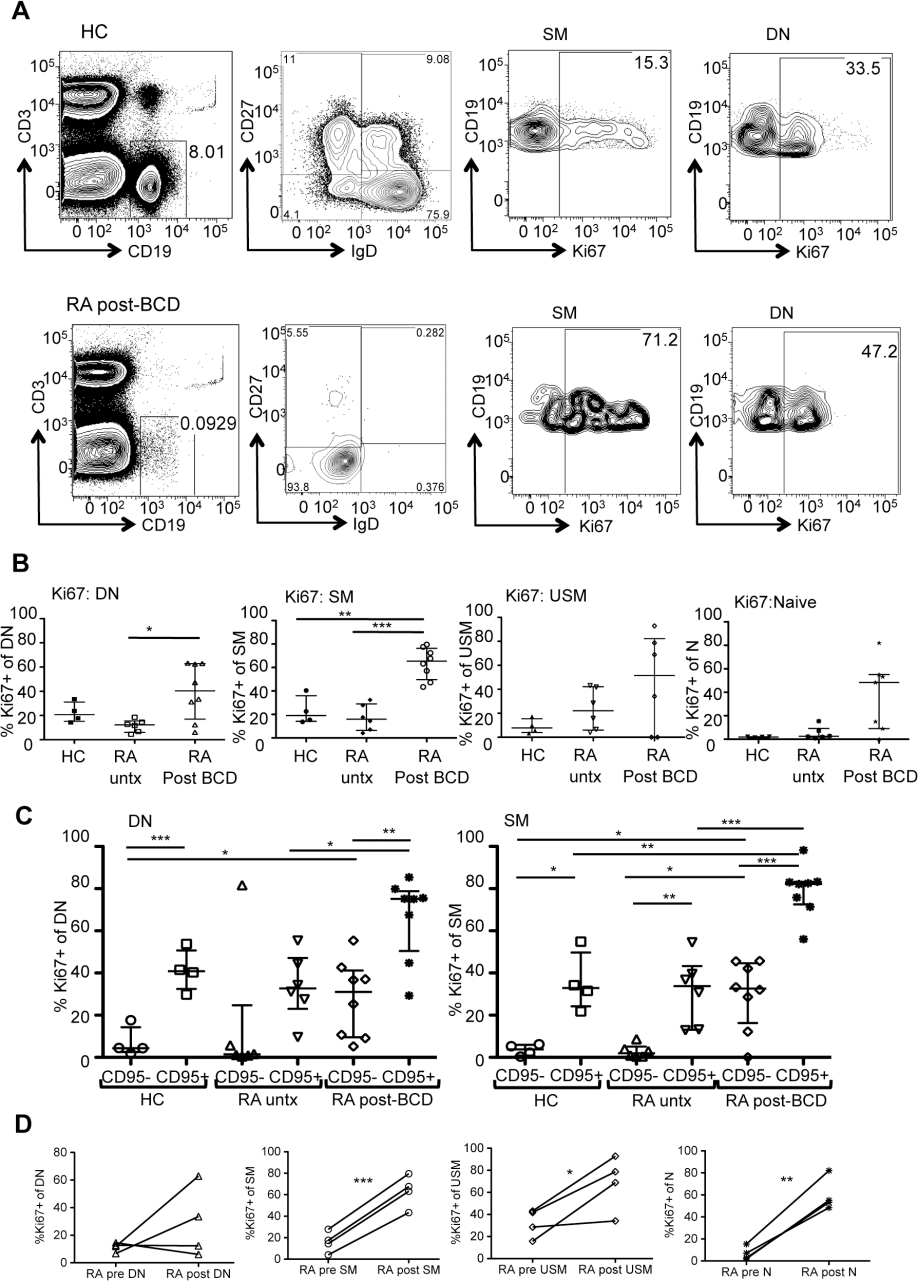
Residual memory B cells in RA following BCDT upregulate Ki67. (A) Dot plots show the expression of intracellular Ki67 (a proliferation antigen) in the gated SM and DN memory B cells. Dramatic up-regulation is seen in the RA patient after BCD compared to a healthy control. (B) Ki67 expression is significantly different in the DN and SM for RA post-BCD (n = 8) compared to untreated RA (n = 6) and healthy controls (n = 4) (*p<0.05, **p<0.005, ***p<0.0005) (Mann-Whitney) (3 group comparison DN p = 0.05, SM p = 0.0017) (Kruskal-Wallis). (C) CD95- B cells have very low expression of Ki67 in HC and untreated RA. Ki67 expression is higher in CD95+ B cells in the healthy controls in all 4 subsets (data not shown for Naïve and USM) (paired t-test). An increase in Ki67 expression is seen post-BCD in both the CD95+ and CD95- populations (*p<0.05, ***p<0.0005) (Mann-Whitney) (DN: 3 group comparison Kruskall-Wallis p = 0.0255 for CD95+ and p = 0.0420 for CD95-; SM: p = 0.0018 for CD95+ and p = 0.0284 for CD95-). The RA subjects post-BCD (n = 8) are B cell depleted (data not shown) and ranged from 3 to 6 months post-treatment. Data is expressed as the median +/- interquartile range. (D) Increase in Ki67 expression after BCD in matched samples (paired t test: *p<0.05, **p<0.005, ***p<0.0005). Post samples are 5 months after rituximab treatment.

To begin to explore whether residual memory B cells may undergo homeostatic proliferation in the lymphopenic environment, we examined the expression of Ki-67, a nuclear antigen that identifies cells recently entering the cell cycle). The SM and DN memory B cell subsets had high expression of Ki-67 during the depleted state as compared to the healthy controls (SM %Ki67+ RA depleted 63.5±4.7% vs. HC 23.0±6.1, p = 0.0017 and DN %Ki67+ RA depleted 39.86±8.1% vs. HC 22.3±4.2, p = 0.05) (mean±SEM) (Fig 3A and 3B).

Ki-67 results suggest homeostatic proliferation of memory B cells after B cell depletion therapy. To ensure that these findings were truly a reflection of BCD and not the RA disease process, we compared an independent cohort of (untreated) RA patients and found no differences from healthy controls (e.g. SM %Ki67+ RA untreated 15.95±4.6% vs. HC 23.0±6.1) (Fig 3B). In contrast, RA post-BCD had significantly higher Ki67 expression compared to untreated RA in both the SM (63.5±4.7% vs. 17.2±4.6, p = 0.005) and DN compartments (39.9±8.1% vs. 11.5±2.1, p = 0.05). Another explanation for these findings could be a shift in the frequency of B cells of varying proliferation states before and after BCDT. Interestingly, CD95+ B cells overall have higher Ki67 expression (Fig 3C) (HC CD95+ vs. CD95-: DN p = 0.005, SM p = 0.0118), with very little expression in CD95- memory B cells from healthy controls and untreated RA. However, an increase in Ki67 expression was seen post-BCD in both the CD95+ and CD95- populations with CD95+ significantly higher than CD95- (DN: p = 0.0027 and SM p = 0.0002), suggesting that elevated Ki67 is related to the B cell depleted state (Fig 3C). Further supporting the conclusion that B cells proliferate following BCD, Ki67 expression significantly increased in multiple subsets (SM, USM, N) in a subset of patients followed longitudinally (Fig 3D).

### Sequential depletion and repopulation of B cells after BCD

**Fig 4:**
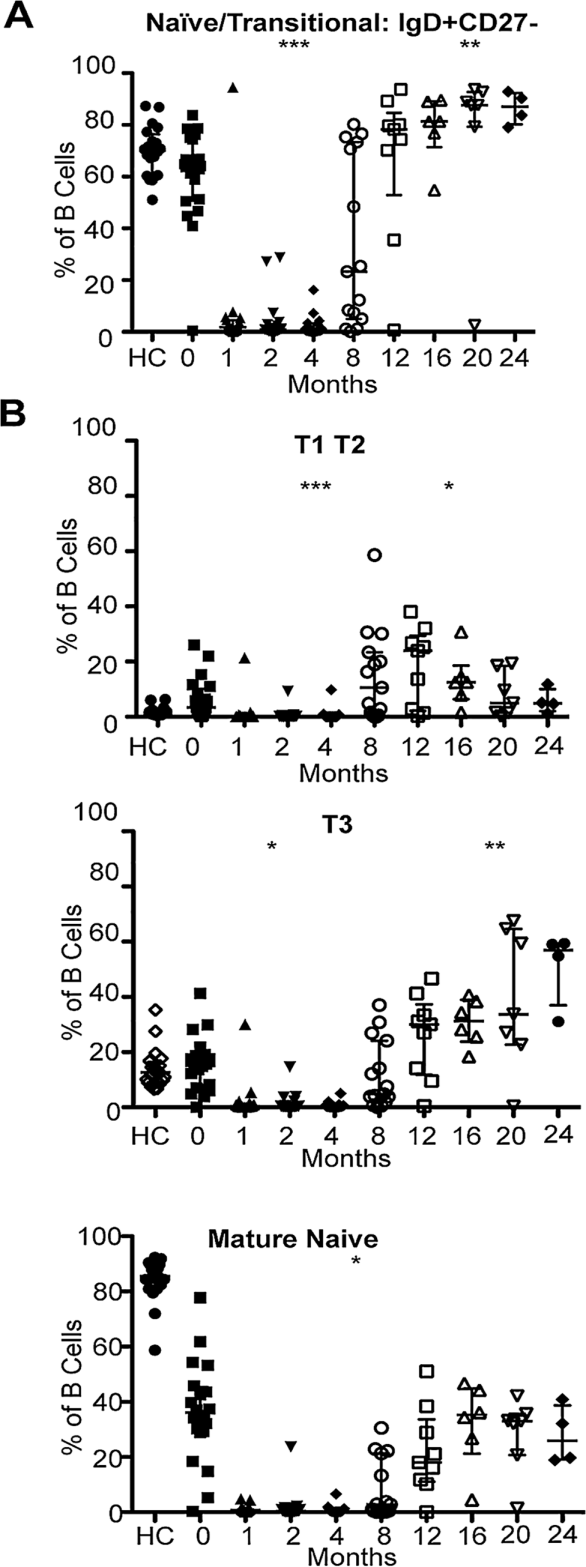
Sequential repopulation of B cells in RA patients after B cell depletion. (A) Naïve/transitional B cell subsets are gated as described as IgD+CD27- (expressed as a % of the total B cell compartment). These B cells are rapidly depleted and remained significantly lower than baseline (***p<0.0001 compared to baseline) until 8 months when reconstitution begins (**reconstitution higher than baseline, p = 0.0137 for 16 month, 0.0062 for 20 month, 0.0037 for 24 month). (B) T1/T2, T3, and mature naïve B cells are gated within the IgD+CD27- population based on MTG, CD38, and CD24 expression and show sequential repopulation. The transitional B cells are rapidly depleted by 1 month and remained significantly lower than baseline (***p<0.0001 compared to baseline) until 8 months when reconstitution begins. The transitional B cells became significantly higher than baseline with reconstitution (*p value: T1/T2 .047916 month 16; T3: 0.0062, 0.0161, 0.0029 at 16, 20, 24 months). The mature naïve significantly decrease at months 1 through 12 (*p ranging from <0.0001 to 0.0426) but re-equilibrate to baseline at months 16 through 24. P values are calculated by Mann-Whitney. Data is expressed as the median +/- interquartile range.

The naïve/transitional compartment (N/T) (IgD+CD27-) was rapidly depleted by 1-month post BCDT in all subjects (<5 cells/μl in all, mean absolute N/T 4.74±17.6, mean total absolute B cells 5.54±18.8) and remained depleted for 4 months (<5 cells/μl) (N/T %: baseline vs. month 1, 2, 4 p<0.0001) (Fig 4). Reconstitution varied between patients beginning between 4 and 16 months (11% of subjects with >5 naïve cells/μl at 4 months, 80% 8 months, 89% by 12 months), with significantly higher naïve/transitional B cell fractions compared to baseline beginning at 16 months (baseline vs. 16 month p = 0.0137, 20 month p = 0.0062, 24 month p = 0.0037) (Fig 4A).

The naïve/transitional subset is composed of T1, T2, T3, and mature naïve B cells [23]. Reconstitution occurred sequentially, with the T1/T2 fraction appearing first, followed by T3, and finally the mature naive. The T1/T2 compartment remained expanded relative to baseline from months 12 through 16 (month 12 p = .0245 and month 16 p = 0.149) and the T3 from month 12 through 24 (12 month p = 0.0426, month 16 p = 0.0132 month 20 p = 0.0386, month 24 p = 0.0029) (Fig 4B).

### Biomarkers of clinical response

**Fig 5:**
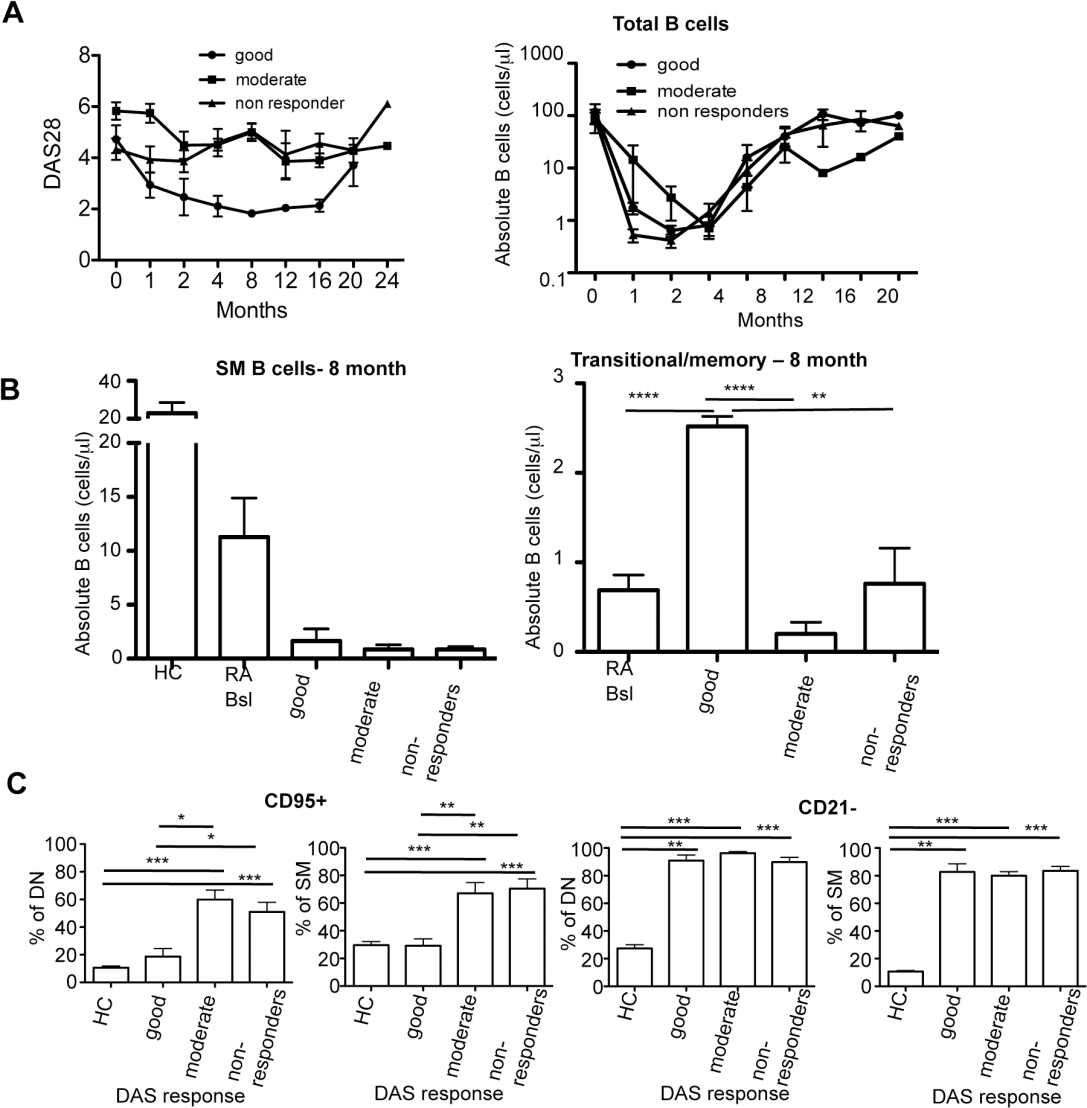
Biomarkers of clinical response. The RA patients are separated into groups based on DAS response at 4 months. (A) The DAS28 is depicted over time in the 3 different clinical response groups. (B) The depletion in total absolute B cells over time is depicted in the 3 different clinical response groups, where globally a similar depth of depletion and kinetics of reconstitution is observed. In the middle graph absolute numbers of switched memory B cells remain low at 8 month post-depletion in all 3 response groups. In the right graph the ratio of absolute numbers of transitional B cells to memory B cells is significant higher in the good responders compared to RA baseline, moderate, and non-responders. (C) CD95+ and CD21- subsets from DN and SM B cells are elevated in non- and moderate responders at 4 months as compared to healthy controls. Notably, the CD95+ from the SM and DN subsets are significantly higher in the moderate and non-responders than the good responders. (Mann-Whitney) (*p<0.05, **p<0.005, ***p<0.0005). Data is expressed as the mean +/- SEM.

As previously reported in the literature, patient response to rituximab was variable. The DAS response patient breakdown at 4 months was as follows: good- 20%, moderate- 35%, and non-responder- 45%. The extent and length of depletion and reconstitution varied from patient to patient. However, there was no clear relationship between the depth of depletion at 1 or 2 months and the clinical response. In our cohort, all subjects depleted to <5 cells/μl, but all still had detectable B cells by high sensitivity flow (lower limit of detection 0.1 cells/μl) (Fig 5 and S1 Fig). Overall, reconstitution occurred with similar kinetics in the good, moderate, and non-responders, as did the reconstitution of SM B cells (Fig 5A, 8 months). Notably, however, the CD95+ subsets of DN and SM were significantly lower at 4 months in the good responders compared to both moderate and non-responders (Fig 5B). The CD21- subsets of SM and DN were not significantly different between responder groups. Baseline levels of CD95+ and CD21- in both the SM and DN B cells also were not predictive of DAS response (data not shown). Notably, the ratio of transitional to memory B cells at 8 months was significantly higher in the good responders (Fig 5A).

### Memory B cells have a greater propensity to produce pro-inflammatory cytokines and impact of B cell depletion therapy

**Fig 6:**
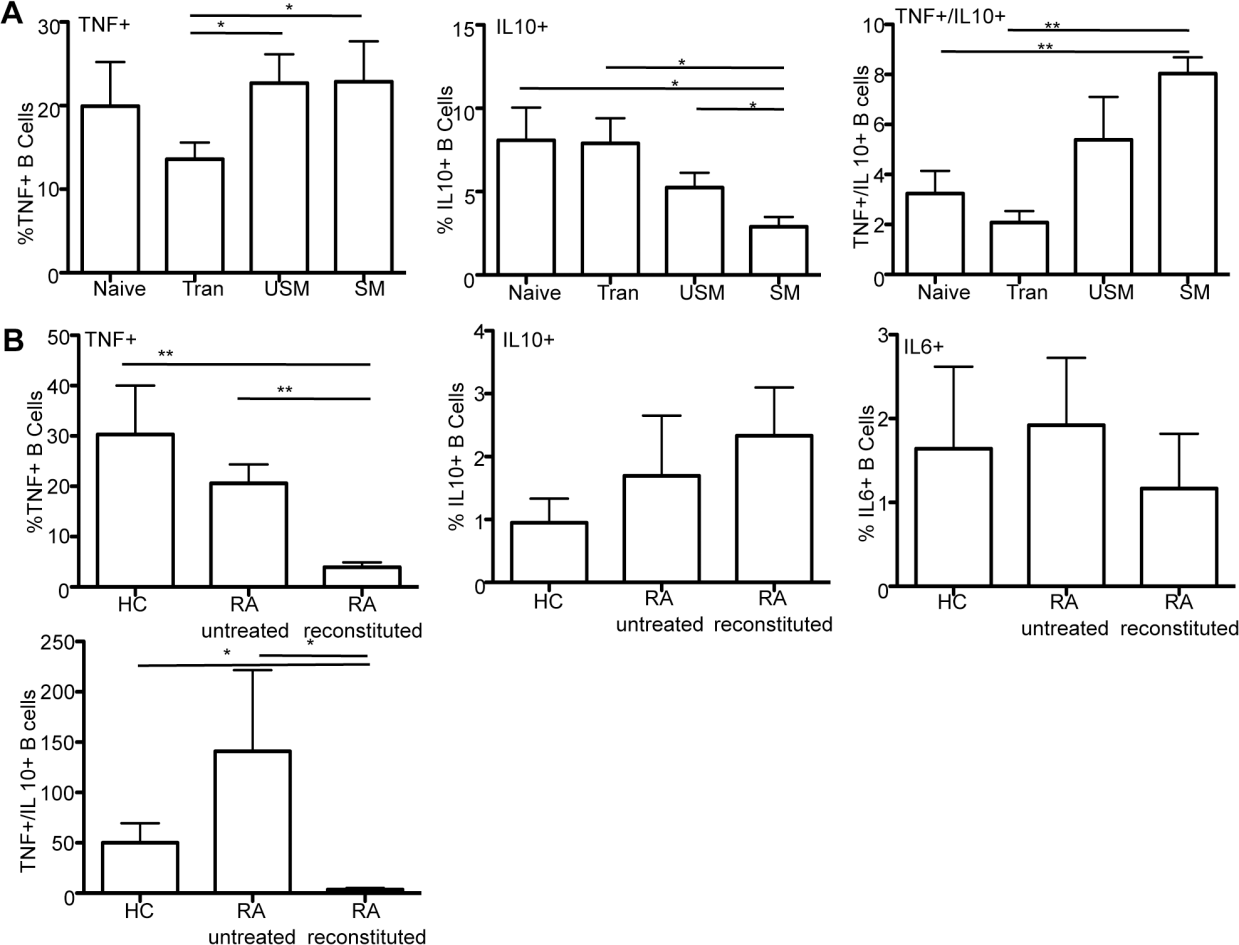
B cell cytokine expression. (A) B cell subsets are sort purified from healthy control peripheral blood and then stimulated for 4 days with 2.5 μg/ml CPG 2006, 2.5 μg/ml anti-CD40, and 50 U/ml IL-2 followed by PMA/ionomycin for 4.5 hours. Intracellular expression of TNF versus IL10 in each subset is quantitated. TNF expression is significantly higher in the USM and SM than the transitional B cells. IL-10 is significantly lower in the SM than in naïve, transitional, and USM B cells. SM B cells have a significantly higher ratio of TNF+ to IL10+ B cells than naïve and transitional (*p<0.05, **p<0.005) (Paired t-test). N = 6 healthy controls. (B) Total PBMCs are stimulated for 4 hours with PMA/ionomycin to compare B cell cytokine expression in untreated RA (n = 6) versus RA after BCD and reconstitution (n = 6) ranging from 15 to 32 months after rituximab (mean = 22 months) versus HC (n = 8). The intracellular expression of TNF, IL10, and IL6 is examined in CD19 gated B cells. B cell expression of TNF is significantly reduced in RA after reconstitution (**p<0.005) (Mann-Whitney). There are no significant differences in IL10 and IL6 producing B cells. The ratio of TNF to IL10 producing B cells is significantly lower in RA after BCD/reconstitution (*p<0.05). Data is expressed as the mean +/- SEM.

Given the variation in depletion of memory B cells and transitional to memory reconstitution observed here, we next asked whether different B cell subsets have distinct abilities to produce cytokines. Initially, B cells from healthy controls were sort purified into naïve, transitional, USM, and SM and cultured for 4 days. The pro-inflammatory cytokine TNF and the anti-inflammatory cytokine IL10 were measured by intra-cellular flow cytometry. Notably, TNF production was higher in the USM (p = 0.0318) and SM (p = 0.05) B cells compared to transitional B cells (Fig 6A). Conversely, the SM compartment produced significantly less IL10 than the transitional (p = 0.0053), naïve (p = 0.0198), and USM (p = 0.0144) B cells. Correspondingly, the ratio of TNF+ to IL10+ B cells was significantly higher in the memory compartment compared to the naïve (p = 0.0065) and transitional (p = 0.0007) compartments.

Next, we analyzed the impact of BCDT on cytokine production (Fig 6B). The ability of RA B cells to produce pro-inflammatory versus anti-inflammatory cytokines in reconstituted patients after BCDT was compared to untreated RA and healthy control after a 4-hour stimulation of PBMCs. All rituximab treated RA patients were under good disease control at the time of the analysis. B cell production of TNF was significantly decreased in reconstituted RA patients as compared to both healthy control (p = 0.0007) and RA untreated (p = 0.0022). This correlated with a shift in B cell phenotype, with more naïve/transitional and less switched memory after B cell depletion/reconstitution (data not shown). B cell production of IL10 and IL6 in RA untreated and RA reconstituted was not different from healthy controls. The ratio of TNF to IL10 producing B cells was significantly lower in RA after BCD and reconstitution as compared to healthy control (p = 0.014) and RA untreated (p = 0.0411) (Fig 6B).

## Discussion

Despite the success of BCDT for the treatment of RA, there is still uncertainty regarding its mechanism of action. Moreover, biomarkers to stratify patients into those most likely to respond to B cell depletion versus other treatment modalities and assess the need for re-treatment are needed. Here we explored how B cell phenotype and function is altered in RA and whether any parameters are predictive of clinical response. At baseline, RA patients had a significant expansion of activated memory B cell populations expressing high levels of CD95 and low levels of CD21 in the peripheral blood. Notably, these populations became dominant post-BCD, suggesting relative resistance to depletion. As suggested by increased expression of Ki67 the CD95+ memory B cells also may be proliferating in the depleted environment. Further, patients with good clinical responses had lower CD95+ activated memory B cells at depletion time points and a higher ratio of transitional to memory B cells at reconstitution. Finally, B cell function is dependent on the B cell subset with memory B cells more prone to pro-inflammatory cytokine production and a significant reduction after BCD.

Although the presence of autoantibodies is an important biomarker of response to BCDT, they are insufficient to predict response and notably do not decline reliably after treatment [37] [38]. In terms of flow cytometry biomarkers, a number of publications have found that the detection of residual peripheral blood B cells using high sensitivity flow cytometry and the return of B cells, especially with higher fractions of memory B cells, increases the risk of inadequate response and/or relapse [24] [21, 25]. For example, Dass *et al*. found that clinical responses at 9 months are predicted by the degree of depletion (63% of RA patients had detectable B cells after the 1st rituximab infusion and 18% after the 2nd infusion), with 82% of patients with complete depletion having a moderate to good EULAR response compared to only 43% of those with detectable residual B cells. In contrast, in our study although every subject depleted to <5 cells/μl, all still had detectable B cells by high sensitivity flow, and overall lower moderate to good responses (44%). Our results suggest that the degree of depletion of activated memory B cells as opposed to total B cells is a more robust biomarker of response.

One question is why these subsets may be more resistant to depletion. Although anti-CD20 is usually relatively effective in depleting B cells from peripheral blood, success in depleting B cells from other sites such as lymph nodes or tertiary lymphoid tissues may be more variable. Thus, Kavanaugh and colleagues found that synovial B cells are decreased but not eliminated by rituximab therapy [39], a result confirmed in other studies [40]. In both these studies higher levels of clinical response correlated with more consistent synovial B cell and plasma cell depletion. Interestingly, it has been reported that the majority of synovial B cells in RA express CD27 [41], and we have similarly found that synovial fluid and tissue B cells are predominantly of a memory phenotype with high CD95 expression compared to the peripheral blood (both SM and DN, data not shown). This is in accord with other literature demonstrating CD21 low B cells enriched in the synovium [42]. Indeed, an alternative explanation for the high expression of Ki67 on B cells after BCD is recirculation of these activated and resistant tissue resident memory B cell populations. However, the fact that Ki67 expression is higher in both CD95+ and CD95- memory B cells after BCD and even in naïve B cells in patients examined before and after treatment argues in favor of homeostatic proliferation. Regardless, these results suggest that the failure to adequately deplete pathogenic B cells in tissue sites is an important predictor of incomplete response to BCDT. This may explain the results of another recent study which found that RA patients with a lower baseline percentage of circulating CD27+ memory B cells had better DAS responses 24 weeks after BCDT [43]. Along similar lines, another study found that an elevated mRNA plasmablast signature at baseline was predictive of reduced clinical response to B cell depletion [44].

Another significant finding of our study is that B cell reconstitution in RA varies, with a higher ratio of transitional to memory B cells a biomarker of good clinical response. This may in part be reflective of better depletion of pathogenic memory B cells and is in accord with the literature that reconstitution with higher memory B cells correlates with earlier relapse of disease [24, 42]. The relationship between clinical response and long term depletion of memory B cells in RA bone marrow has also been suggested [45] [46]. Faster memory B cell and plasmablast reconstitution also correlates with early relapse in SLE [47]. However, we also contend that the therapeutic effect of B cell depletion does not lie solely in the depletion of B cells since not all of the effects of B cells promote autoimmunity. In particular, our results indicate that the transitional to memory B cell ratio at 8 months as opposed to the total absolute numbers of memory B cells is a marker of clinical response, suggesting that the outcome of B cell depletion depends on the balance between protective and pathogenic B cell populations.

One of the ways in which B cells can exert effects on other immune cells is via cytokine secretion. From an autoimmunity standpoint, cytokine production by B cells may either stimulate or inhibit pathogenic responses. Thus, B cells are able to suppress autoimmunity in different animal models either through the production of IL10 or TGFβ as well as by cytokine independent functions. In mice, this function appears to be mediated by a discrete subset of B cells, variably identified as CD1dhi/CD21hi/CD23hi transitional-marginal zone precursors [48] or CD1dhi/CD5+ B10 cells [49], and termed B regulatory cells. B regulatory cells are protective against murine inflammatory arthritis in part via the production of IL10 [49] and are induced after BCDT in a murine autoimmune diabetes model [50]. In humans, CD24hiCD38hi B cells have been identified [23] with similar properties, including the ability to secrete IL10, inhibit T cell proliferation, and T cell cytokine release [18, 51]. An intriguing recent study found that this regulatory subset of B cells maintains regulatory T cells, negatively regulates Th17 cells, and is defective in active RA [26]. Whether this defect is corrected after BCD is a major question. Our results demonstrate that CD24hiCD38hi B cells are higher after BCD and reconstitution particularly in good responders, but IL10 production by B cells did not vary significantly. Notably, however, the ratio of TNF to IL10 producing B cells is significantly lower after BCD, suggesting that at the individual patient level the balance of pro-inflammatory versus anti-inflammatory B cell cytokine production is shifted. A study in MS demonstrated that IL10 production is impaired in peripheral B cells of patients with active MS, a defect that is reversed after B cell depletion therapy [20]. We suggest that this effect is likely mediated by a shift in predominant B cell populations present, with variable propensity for IL10 versus pro-inflammatory cytokine secretion.

Importantly, we found that TNF production by B cells is dramatically reduced after B cell reconstitution, correlating with a reduction in memory B cells. As noted before cytokine production by B cells may inhibit or stimulate pathogenic autoimmune responses. An example of the latter is the participation of B cells in ectopic lymphoid tissue formation and maintenance [52], supported by the finding that these tissues do not efficiently develop in B cell deficient mice [53]. Furthermore, depleting B cells in synovial biopsies from RA patients caused diminishment of the ectopic follicles and reduced the autoreactive T cell response in SCID mice transplant models [12]. Notably, cytokine-producing B cells (IFNγ, IL6, TNFα, LTα) are found directly within these ectopic lymphoid tissues [53, 54]. While our study does not directly address synovial B cells, it is likely that a shift in B cell phenotype and function after B cell depletion and reconstitution disrupts ectopic lymphoneogenesis.

Another recent study found that MS patient B cells produced increased IL6 compared to healthy controls, an abnormality normalized with B cell reconstitution after rituximab. Interestingly, in mice B cell derived IL6 potentiated Th17 cells [19], prompting the authors to speculate that B cell depletion ameliorates autoimmunity in this setting through ablation of IL6-producing B cells. However, in our hands IL6 producing B cells are not different in active RA compared to reconstituted patients and healthy controls. This may be related to differences in the two diseases and/or the methods used for analysis (both type of stimulation and detection vary). Regardless, we speculate that pro-inflammatory cytokine production by memory B cells in RA (whether TNF or IL6) may promote T cell activation and Th17 differentiation. This could be balanced by the function of other B cell subsets, including transitional and naïve, with a propensity for IL10 production, potentiation of T regulatory cells, and inhibition of pathogenic Th17 responses.

## Conclusions

Although B cell activation presumably occurs in target organs such as the synovium in RA, it is notable that active disease is associated with significant peripheral blood expansion of activated memory B cell populations. These populations may be resistant to B cell depletion as they become dominant after treatment, which likely has critical implications for treatment response given that good clinical outcomes were associated with lower CD95+ activated memory B cells at depletion time points and a higher ratio of transitional to memory B cells at reconstitution. We speculate that alterations in B cell phenotype and corresponding function are critical to the mechanism of action of BCD given the accompanying shift in B cell pro-inflammatory cytokine production. Overall, our results add to the emerging data that the clinical and immunological outcome of BCD depends on the relative balance of protective and pathogenic B cell subsets established after B cell depletion and upon B cell reconstitution.

## Supporting Information

S1 Fig Absolute B cell populations following rituximab treatment. (A) Change in SM and DN memory over time after BCDT for all subjects. In this graph the points in color represent the 3 patients who received rituximab previously. (B) Kinetics of change in the cohort as a whole for CD95 and CD21 expression. (C) Absolute numbers of naïve/transitional B cells and mature naïve (excluding transitional) over time. Data is expressed as the median +/- interquartile range. If a data point is missing absolute numbers were not available at that time point.
(TIF)
