# Supplementary figures and images for: Expansion of Activated Peripheral Blood Memory B Cells in Rheumatoid Arthritis, Impact of B Cell Depletion Therapy, and Biomarkers of Response
Source: PLoS One. 2015 Jun 5;10(6):e0128269. doi: 10.1371/journal.pone.0128269 (PMC4457888; doi:10.1371/journal.pone.0128269)

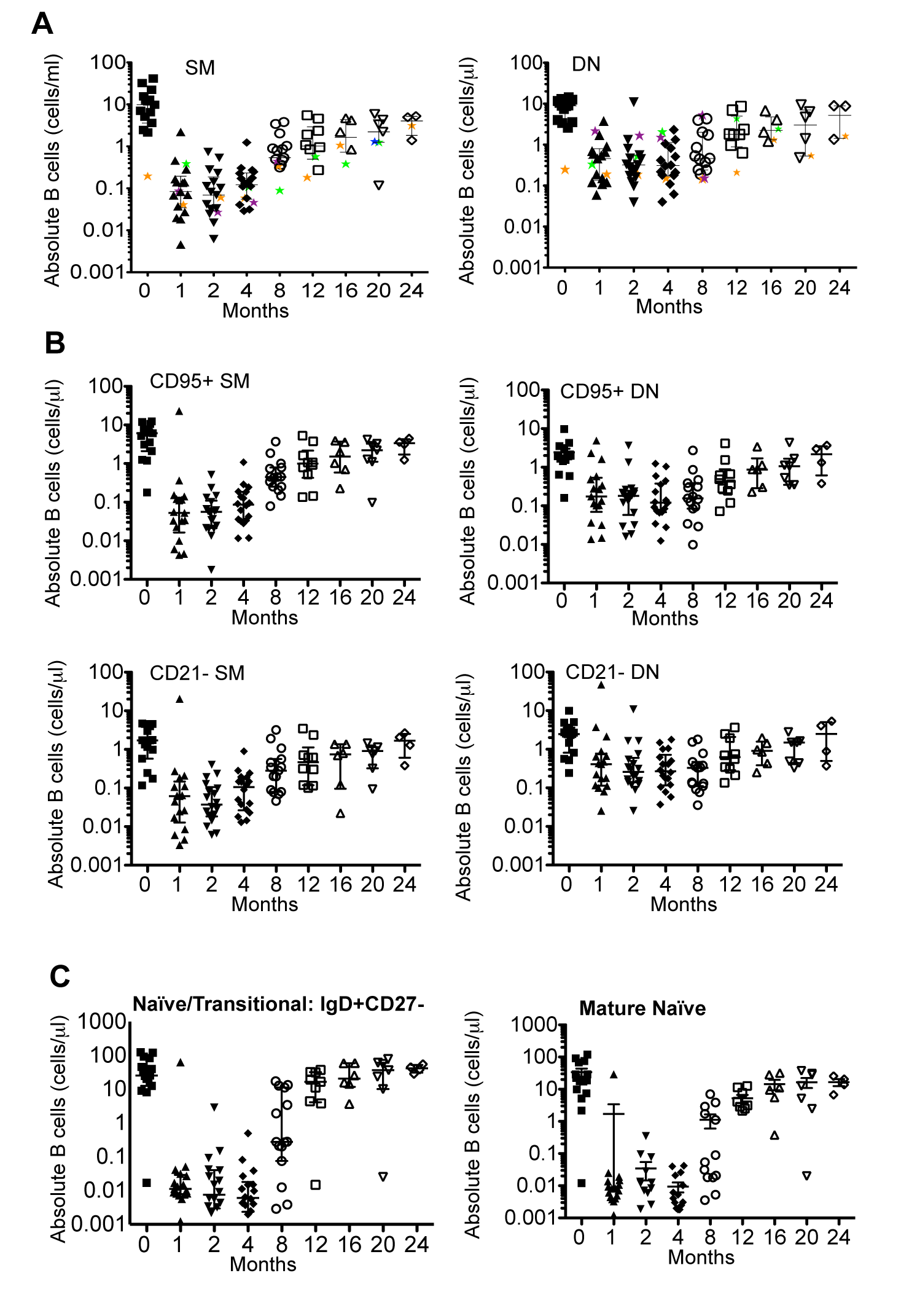

Supplement: S1 Fig — (A) Change in SM and DN memory over time after BCDT for all subjects. In this graph the points in color represent the 3 patients who received rituximab previously. (B) Kinetics of change in the cohort as a whole for CD95 and CD21 expression. (C) Absolute numbers of naïve/transitional B cells and mature naïve (excluding transitional) over time. Data is expressed as the median +/- interquartile range. If a data point is missing absolute numbers were not available at that time point. (TIF) [file pone.0128269.s001.tif]
